# Circ0007042 alleviates intervertebral disc degeneration by adsorbing miR-369 to upregulate BMP2 and activate the PI3K/AKt pathway

**DOI:** 10.1186/s13075-022-02895-7

**Published:** 2022-09-06

**Authors:** Zhenyu Wang, Yuguang Zhao, Yi Liu, Zhigang Qu, Xinming Zhuang, Qingxu Song, Haoyu Li, Jiali Leng

**Affiliations:** 1 Department of Spinal Surgery, the First Hospital of Jilin University, Changchun, China; 2 Cancer Center, the First Hospital of Jilin University, Changchun, China; 3 Department of Hospice, the First Hospital of Jilin University, No.1 Xinmin Street, Changchun, Jilin, 130021 People’s Republic of China

**Keywords:** Intervertebral disc degeneration, circ_7042, miR-369-3p, CDH2, BMP2

## Abstract

**Background:**

To identify regulatory ncRNA molecules that can cause differential expression of CDH2 in intervertebral disc degeneration (IDD) and explore whether there are other ways to affect the progression of IDD.

**Methods:**

A primary culture of human nucleus pulposus (NP) cells was established and identified by immunofluorescence. An in vitro IDD model was constructed by compressing human NP cells, and the MTT assay was used to measure cell viability. Changes in the ncRNA group were analysed by RNA-seq. The expression levels of hsa_circ_7042, CDH2, and miR-369-3p were detected by qPCR. Cell apoptosis, senescence, and extracellular matrix (ECM) metabolism were detected by flow cytometry, β-galactosidase staining, and Western blotting. hsa_circ_7042, miR-369-3p, and bone morphogenetic protein 2 (BMP2) were verified by luciferase and RNA immunoprecipitation (RIP) analyses. The PI3K/Akt pathway was validated by transfection of BMP2 siRNA. Furthermore, a mouse model of lumbar spine instability was constructed. circ_7042 adenovirus was packaged and injected into the intervertebral discs of mice, and the influence of circ_7042 overexpression on intervertebral disc degeneration was determined.

**Results:**

Western blotting, qPCR, and flow cytometry analyses confirmed that overexpression of circ_7042 could downregulate miR-369-3p and upregulate the expression of CDH2 and BMP2 in IDD cell and animal models. Additionally, the levels of apoptotic and senescent cells decreased, and ECM degradation decreased. The PI3K/Akt pathway was significantly activated after circ_7042 was overexpressed. The injection of circ_7042-overexpressing adenovirus effectively reduced ECM degradation and the level of apoptosis in NP tissue.

**Conclusions:**

circ_7042 could upregulate the expression of CDH2 and BMP2 by absorbing miR-369-3p, and the increased BMP2 activated the PI3K/Akt pathway, thus improving IDD.

**Supplementary Information:**

The online version contains supplementary material available at 10.1186/s13075-022-02895-7.

## Background

Intervertebral disc degeneration (IDD) occurs in animals capable of upright walking and is an age-related disease. Almost all people will have various degrees of IDD during their ageing process. In terms of histology, the formation of IDD is characterized by the initial dehydration and shrinkage of the nucleus pulposus (NP), which gradually develops into annular fissure, ossification of endplate cartilage, annular breakage, and finally prolapse of the NP and severe loss of intervertebral disc function [1]. Therefore, NP lesions are an important initiating factor of IDD. However, the aetiology of IDD is very complex and is related to the patient’s age, immune system physiology, living habits, and other factors. Pathological studies have confirmed that IDD is mainly caused by physical factors, biochemical conditions of the microenvironment of NP cells, and inflammatory stimulation [1, 2]. However, there is little understanding of the pathophysiological changes associated with IDD, especially the molecular biology of NP degeneration, which is an important initiating factor of IDD. Therefore, it is of great significance to reveal the molecular and pathogenic mechanisms of IDD.

N-cadherin (CDH2) is the first adhesion molecule found in the nervous system [3]. Recent studies have found that CDH2 is also highly expressed in normal NP cells and is gradually downregulated with the development of intervertebral disc degeneration [4, 5]. Meanwhile, NP cells can induce senescence under high compressive stress, and N-cadherin (CDH2) expression can significantly delay the senescence of NP cells [6]. In addition, CDH2-mediated signalling contributes to the maintenance of normal NP cell phenotypes and NP matrix biosynthesis under the stimulation of physical intervertebral disc injury factors [7, 8]. However, the regulatory mechanism of CDH2 is not well understood.

ncRNA is a very stable type of ncRNA that often exhibits a bidirectional interaction and network type of stable expression regulation. miRNA is a small RNA approximately 20–24 nucleotides in length that accounts for a large proportion of ncRNA and affects more than 30% of gene expression in cells through multiple pathways. In the occurrence and development of IDD, miRNAs are involved in the progression of the disease by acting on apoptosis, the inflammatory signal response, and extracellular matrix (ECM) components. Studies have shown that miR-369-3p can affect the expression of CDH2. For example, in a study of neurons, overexpression of miR-369-3p significantly inhibited the expression of CDH2 and thus inhibited the differentiation and migration of neurons [9]. Furthermore, miR-369-3p affected CDH2 function by downregulating ADAM10 expression of metalloproteinase in cortical development [10]. These studies suggest that mir369-3p could influence CDH2 function through direct and indirect means. In addition to common miRNAs, ncRNAs also include LncRNAs and circRNAs, which participate in gene regulation [11]. circRNAs are a class of very stable circular ncRNAs. In addition to regulating mRNA levels, circRNA can also constitute an “RNA sponge” in the ncRNA regulatory network and competitively antagonize miRNA functions through adsorption of miRNA, thereby affecting the expression of miRNA-targeted mRNAs [12]. Recent studies have revealed that circRNAs can also play an important role in the development of IDD. For example, circRNA_CIDN alleviates compressive load-induced injury of human NP cells through the miR-34a-5p/SIRT1 axis [13]. The circRNA circ_GRB10 inhibits the disease progression of human disc degeneration [14], and the circRNAs ITCH [15] and GLCE [16] can regulate the function of NP cells by interacting with miRNA. The above studies have proven that circRNAs play an important role in the pathogenesis of IDD.

In this study, NP cells showed senescence under compression stress, and CDH2 expression was significantly downregulated. By comparing normal NP and analysing the difference in circRNA, it was found that the downregulation of the hsa_circ_7042 gene may reduce the adsorption of miR-369-3p. This results in downregulation of CDH2 in NP cells, and it was further confirmed in the mouse IDD model that overexpression of circ_7042 can improve IDD through the upregulation of CDH2. The results of this study suggest that regulating circRNA expression may be a potential treatment for IDD.

## Methods

### Primary culture of human NP cells

Human NP tissue (sample from the First Hospital of Jilin University, donor signed informed consent) was washed with PBS and cut into approximately 1 mm^3^ pieces with a scalpel. First, tissue was treated with 0.25% trypsin (Gibco, USA) at 37 °C for 0.5 h, followed by 0.2% type II collagen enzyme (Invitrogen USA) for another 3 h at 37 °C [13]. The cell suspension was centrifuged, and the supernatant was discarded. The cell precipitates were resuspended in DMEM/F12 (Gibco, USA) complete medium containing 15% FBS (Gibco, USA) and 1% penicillin–streptomycin (HyClone, USA). The cells were cultured at 37 °C in a 5% CO_2_ incubator. When NP cells grew to confluence, they were digested with 0.25% trypsin and passaged. P2–P3 cells were used for subsequent experiments.

### Primary cell identification

Immunofluorescence was used to identify the expression levels of aggrecan (cat no. A11691) and collagen II (cat no. A1560) in human NP cells. Refer to the immunofluorescence experiment for specific details.

### Compression treatment

Human NP cells were seeded in a 6-well culture plate prepositioned with cover glasses. The plate was then placed in a stainless steel pressure vessel with a pressure gauge and pumped with a mixture of 0.5% carbon dioxide and 99.5% compressed air. When the static pressure in the container reached 1.0 MPa, the container was transferred to an incubator at 37 °C. The cells received static compression for 36 h, unless otherwise noted.

### HE staining

Cover slides were placed in 6-well plates, and 1×10^6^ cells were added. Cell slides were removed after experimental CT and treatment in different groups for 36 h. After fixation with 4% paraformaldehyde at room temperature for 20 min, a 0.5% Triton X-100 (in PBS) solution was added for 10 min to permeabilize the membranes. Haematoxylin dye was added for 1 min, followed by 1% hydrochloric acid alcohol differentiation. Sodium bicarbonate (0.1%) was used to turn the cells blue, then they were dyed with eosin for 5 s, and finally, 95% alcohol was used to dehydrate and dry the sections, which were then soaked in xylene for 3 min. Neutral gum was added dropwise to seal the cover glasses. The results were observed and photographed by a light microscope (DM500, Leica).

### Detection of MTT cell activity

A 200-μL cell suspension (containing 8000 cells) was inoculated into 96-well plates. The control group was typically cultured under atmospheric pressure, while the model group was cultured under pre-CT. Then, the MTT assay was performed after incubation at 37 °C for 12, 24, 36, and 48 h. MTT (10 μL, 5 mg/mL) was added to each well, and the plates were incubated at 37 °C for 4 h. The medium was removed, 150 μL of dimethyl sulfoxide (DMSO, Sigma) was added to each well, and the container was shaken for 5 min. A Molecular Devices plate reader (USA) was used to measure the absorbance at 560 and 630 nm as a reference wavelength. Three wells were set at each time point.

### Western blot

We treated 5×10^5^ cells or 100 mg of tissue with RIPA lysis buffer. The samples were incubated at 4 °C for 10 min and centrifuged at 10,000×*g* for 15 min at 4 °C. The supernatant was transferred, mixed with sample buffer, and boiled for 5 min. Proteins were isolated by SDS–PAGE electrophoresis and transferred to PVDF membranes (Bio-Rad, USA). The membranes were blocked with 5% skim milk powder for 1 h at room temperature, followed by the addition of primary antibody. Rabbit anti-human cleaved caspase-3 (cat no. 9661), total caspase-3 (cat no. 9662), Akt (cat no. 4691), p-Akt (cat no. 4060), and PI3K (cat no. 4249) antibodies were purchased from Cell Signaling Technology (CST). Antibodies against MMP-13 (cat no. 49328, Signalway Antibody), p-PI3K (cat no. ab278545, Abcam), Bax (cat no. A7626), Bcl-2 (cat no. A0208), collagen II (cat no. A1560), aggrecan (cat no. A11691), MMP-3 (cat no. A11418), CDH2 (cat no. A19083), bone morphogenetic protein 2 (BMP2) (cat no. A0231), and the reference protein GAPDH (cat no. A19056) were purchased from the ABclonal Company. The membranes were incubated with the primary antibodies overnight at 4 °C. The membranes were washed with TBST buffer solution and incubated with goat anti-rabbit IgG-HRP secondary antibody (cat no. AS014, ABclonal) for 1 h. After TBST washing, the bands were exposed to ECL luminescence solution. Exposure was measured using a UVP instrument (ChemiDoc-It Imaging System, CA, USA).

### RT–PCR

The TRIzol method was used to extract the total RNA of lentivirus-coated cells, and the RNA was reverse transcribed into cDNA using a reverse transcription kit (Novoprotein, China). The hsa_circ_7042/mmu_circ_7042, CDH2/CDH2, and BMP2/BMP2 levels were tested according to the instructions of the NovoStart® SYBR qPCR SuperMix Plus kit (Novoprotein, China). Expression of the Acan and Col2a1 genes was detected, and GAPDH/GAPDH was used as an internal control. miR-369 was obtained by following the instructions of the one-step miRcute miRNA kit (Qiagen, Germany). Using U6 as an internal reference, the expression of miR-369 was detected using TransScript® Green miRNA Two-Step qRT–PCR SuperMix (TransScript, China). The formula 2^−ΔΔCt^ was used to determine the relative expression levels of genes, and each sample was repeated 3 times. The qPCR primer sequences are as follows: see Supplementary material 1, Table S1.

### Lentivirus infection

The hsa_circ_7042 is a splicing product of the NCEH1 gene and consists of its second and third exon sequences. PCR was performed to amplify the has_circ_7042 RNA gene, and the *Mlu*I and *Xho*I restriction sites were added to the 5′ ends of the upstream and downstream primers, respectively. The primer sequences were as follows: see Supplementary material 1, Table S2.

PCR products and pLV-circ-GFP-puro plasmids were digested by *Mlu*I (cat no. R0198, NEB cloner, USA) and *Xho*I (cat no. R0146, NEB cloner, USA) restriction enzymes. T4 DNA ligase (D7006, Beyotime) was used to ligate the digestion products. The positive clones were screened and sent for sequencing. After the correct sequence was verified, the recombinant plasmid pLV-circ_7042 was extracted and used. The Zhejiang Ruyao Biotechnology Co., Ltd. was commissioned to pack the lentivirus. After mixing 10 μL of the concentrated virus with 5 mL of complete culture medium, it was added to the target cells to be infected, and 2 μg/mL polybrene was used to enhance the infection efficiency. Forty-eight hours after infection, the GFP fluorescence intensity and ratio were observed under an inverted fluorescence microscope (IX70, Olympus) to determine the infection efficiency. When a large amount of GFP fluorescence was observed under a fluorescence microscope, puromycin containing 2 μg/mL was added for screening, and cell growth was maintained for 7–9 days. Then, the medium was replaced with medium without puromycin for subsequent analysis. In the results, circ_7042 represented the overexpression of hsa_circ_7042.

### Cell cotransfection

The miR-369 mimic, miR-369 inhibitor, and their control were synthesized by Genewiz, and the nucleotide sequences were annealed to form double chains for subsequent cotransfection. For the siRNA design of BMP2, first, the CDS region sequence of BMP2 mRNA was obtained from NCBI, and then the CDS region siRNA was designed by siDirect 2.0 software. Lipofectamine 3000 (Thermo Fisher, USA) was used to transfect the above oligonucleotide fragments into target cells, and the transfection efficiency was determined by qPCR. The siRNA sequences designed were as follows: see Supplementary material 1, Table S3.

### β-galactosidase senescence detection

The cells of different treatment groups were removed from the culture environment. After discarding the culture medium, a fixation solution (1.8% formaldehyde and 0.05% glutaraldehyde, prepared with PBS) was added and fixed for 5 min. Then, PBS was used to wash the cells three times for 5 min each. A staining solution (5 mM potassium ferricyanide, 5 mM potassium ferricyanide, and 2 mM MgCl_2_ prepared with PBS) containing 1 mg/mL x-gal (cat no. X917727, Macklin, China) was added, and the cells were incubated at 37 °C overnight. After the cells were washed with PBS, they were observed and photographed under an inverted microscope. β-Galactosidase-positive cells, which were senescent, appeared blue.

### Bioinformatics prediction

The targeted circRNA of miR-369-3p was predicted by cirBank (http://www.circbank.cn/) and screened for differences in circRNA expression between disc CT status and expected status. It is predicted that hsa_circ_7042 may be the primary circRNA binding to miR-369-3p. ENCORI (http://rna.sysu.edu.cn/encori/rriPathways.php) was used to forecast the miR-369-3p downstream target mRNA associated with intervertebral disc degeneration in Genecard gene screening.

### Double luciferase activity

The circ_7042 and BMP2 3′UTR sequences containing wild-type and mutated miR-369-3p binding sites were cloned into the luciferase vector pIS0 (#12178, Addgene, XYbio, China). circ_7042-WT, circ_7042-MUT, BMP2-3′UTR-WT (WT), and BMP2-3′UTR-MUT (MUT) were generated. The constructed luciferase vector was transfected with PRL-TK vector (Promega) and miR-NC and miR-369 mimic (Genewiz synthesis). After 48 h of transfection, the dual-luciferase reporter gene assay system (Promega) was used. Amplified primer sequences are shown in Supplementary material 1, Table S4.

### RNA pull-down

Cell transfection was performed when NP cells were more than 60% confluent. For transfection, 50 nM biotin-labelled miR-369-3p mimic or miR-NC was mixed with Lipofectamine® RNAi MAX (Invitrogen), and the mixture was added to the cell culture drop by drop. Then, the cells were harvested after 24 h of transfection and lysed. Streptavidin magnetic beads (cat no. AE01, Shanghai Emerther Biotechnology Co., LTD.) were added to the lysate and incubated for 3 h at RT. RNA interacting with miRNA was extracted with TRIzol reagent. The abundances of circ_7042 and BMP2 were detected by agarose gel electrophoresis and qPCR.

### Immunofluorescence

Cells grown on coverslips were taken from each group and fixed with 4% paraformaldehyde at room temperature for 15 min. Triton X-100 (0.5%, dilution in PBS) was added and allowed to permeabilize the cells for 10 min. The cells were then blocked with 1% BSA for 30 min. Rabbit anti-human CDH2, anti-aggrecan, and anti-collagen II antibodies (1:50 dilution) were added and incubated at 4 °C overnight. After washing with PBS, goat anti-rabbit IgG-FITC (SA00002-2, Protetech, China, 1:200 dilution) was added and incubated at room temperature for 1 h. After DAPI staining, the coverslips were sealed with an anti-fluorescence quenching agent (Solarbio, China). The cells were photographed under a fluorescence microscope (DM500, Leica).

### Apoptosis detection

Human NP cells from different treatment groups were collected, and apoptosis levels were determined using an Annexin V-FITC/PI apoptosis detection kit (BD, USA). According to the kit instructions, the cells were collected and washed twice with precooled PBS. The cell precipitates were suspended in 500 μL of binding buffer containing more than 10^5^ cells. Annexin V-FITC (5 μL) and propidium iodide (PI) (5 μL) were added and incubated at room temperature away from light for 15 min. After passing the cells through a 70-μm nylon mesh cell strainer, Attune focused flow cytometry (Life, USA) was performed for data collection and analysis. All samples were evaluated 3 times in triplicate.

### Construction of a mouse model of intervertebral disc degeneration

A mouse model of lumbar spine instability (LSI) was established based on the method of Zheng et al. [17]. Two-month-old male C57BL/6J mice (*n*=24) were used in this study. Anaesthesia was performed with 2% phenobarbital sodium (35 mg/kg). The supraspinal and interspinous ligaments of the L3–L4 lumbar vertebrae were resected to induce lumbar instability. The mice were euthanized by the injection of phenobarbital sodium after 8 weeks of treatment. The animal ethics committee approved this study protocol. The study followed the ARRIVE guidelines [18].

### Adenovirus injection therapy

Adenoviral plasmids for overexpression of circ_7042 were constructed. Adenovirus with a titer of 1×10^8^ TU/mL was provided by the Zhejiang Ruyao Biotechnology Co. Ltd. The mice were randomly divided into 3 groups (*n*= 8 for each group), namely, the normal control group, model group, and circ_7042 group. After the LSI operation, mice in the latter two groups were given an intradiscal injection of 50 μL of adenovirus every 3 days. The experiment ended at week 8. Intervertebral disc tissues were isolated, and the expression levels of the circ_7042, miR-369-3p, Bmp2, Cdh2, Acan, and Col2a1 genes in the tissues were detected by qPCR. Western blotting was used to detect the expression levels of apoptosis- and collagen-related proteins, as well as the PI3K/Akt pathway.

### Pathological detection

The intervertebral disc tissues of L3–L4 were fixed in neutral formaldehyde solution for 24 h and then decalcified with EDTA-Na_2_ solution for 4 weeks. After decalcification, the tissue was dehydrated by gradient concentrations of alcohol and paraffin embedded after clearing with xylene. The tissue was sliced into 5 μm slices, dried in an oven, and set aside. For HE staining, paraffin sections were melted in xylene. After tissue rehydration with gradient alcohol, haematoxylin-eosin staining was performed. After dehydration with 95% alcohol, the tablets were soaked in xylene for 3 min and sealed with neutral gum.

### Safranine O-bright green staining

After paraffin sections were dewaxed in water, haematoxylin staining was performed for 3 min, followed by differentiation with 1% alcohol hydrochloride. After the tap water turned blue, the cells were dyed 0.01% solid green for 3 min. Glacial acetic acid (1%) was used to wash the residual concrete green dye. Safranine O (0.1%) dye was added for 3 min. The residual safranine O dye was washed and dehydrated by soaking in 95% alcohol for 3 min on a xylene transparent neutral gum sealing sheet. A light microscope was used to observe the results.

### Statistical analysis

SPSS 19.0 statistical software was used. All experiments were repeated three times, and the measurement data are expressed as (x±s). One-way analysis of variance (ANOVA) was used for comparisons between multiple groups. *P* <0.05 was considered statistically significant.

## Results

### Constructing the intervertebral disc degeneration cell model

**Fig. 1:**
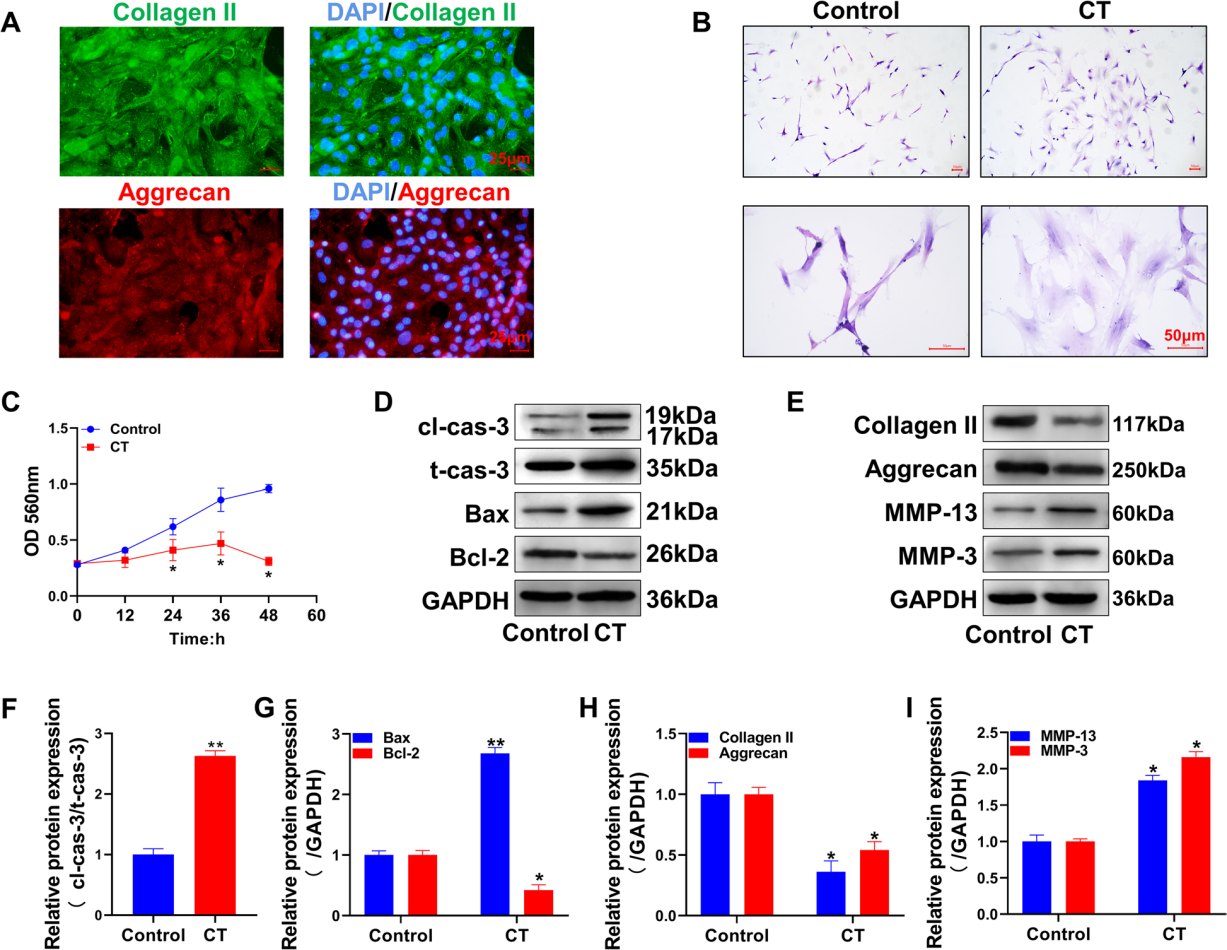
Establishment of the NP cell degeneration model. Collagen II and aggrecan protein expression levels in primary cells were detected by immunofluorescence. **B** Cells were stained with HE. **C** MTT was used to detect cell activity. **D** The expression levels of cleaved caspase-3 (cl-cas-3), total caspase-3 (t-cas-3), Bax, Bcl-2, and apoptosis-related proteins were detected by Western blotting. **E** The protein expression levels of collagen II, aggrecan, MMP-13, and MMP-3 were detected by Western blotting. **F**, **G** show the statistical quantification results of the optical density values of the bands in the Western blotting results in **D**. **H**, **I** show the statistical quantification results of the optical density of the bands in the Western blot results in **E**. *P<0.05, ** *P<*0.01, compared with the control group

The primary culture was successfully obtained through human intervertebral disc tissue culture. The expression of collagen II and aggrecan, biomarkers of human NP cells, was detected by immunofluorescence, as shown in Fig. 1A. The above two proteins showed positive expression in the primary cells. Subsequently, HE staining was performed on the cells after compression treatment (CT). As shown in Fig. 1B, the NP cells in the control group had a smaller cell size and abundant cytoplasm, while in the CT group, the morphology of the cells became larger, the cytoplasm was loose, and the cells were flattened and spread out. The MTT assay was used to measure cell viability (Fig. 1C). The results showed that the cell proliferation in the CT group was significantly lower than that in the control group (*P<*0.05). Figure 1D and E show the expression levels of apoptosis- and collagen-related proteins in each group, respectively, by Western blotting. The results showed that after CT, the expression levels of the proapoptotic proteins cleaved caspase-3 and Bax in the CT group were significantly higher than those in the control group. The expression level of the antiapoptotic protein Bcl-2 was inhibited considerably, with statistically significant differences (*P<*0.05, Fig. 1F, G). Compared with the control group, the protein expression levels of collagen II and aggrecan proteins in NP cells were also significantly decreased in the model group (*P<*0.05). In contrast, the expression levels of the collagen degradation-related proteins MMP-13 and MMP-3 were significantly increased (*P<*0.05), and the differences between groups were statistically significant (*P<*0.05).

### Effect of circ_7042 overexpression on NP cell senescence in a compression environment

**Fig. 2:**
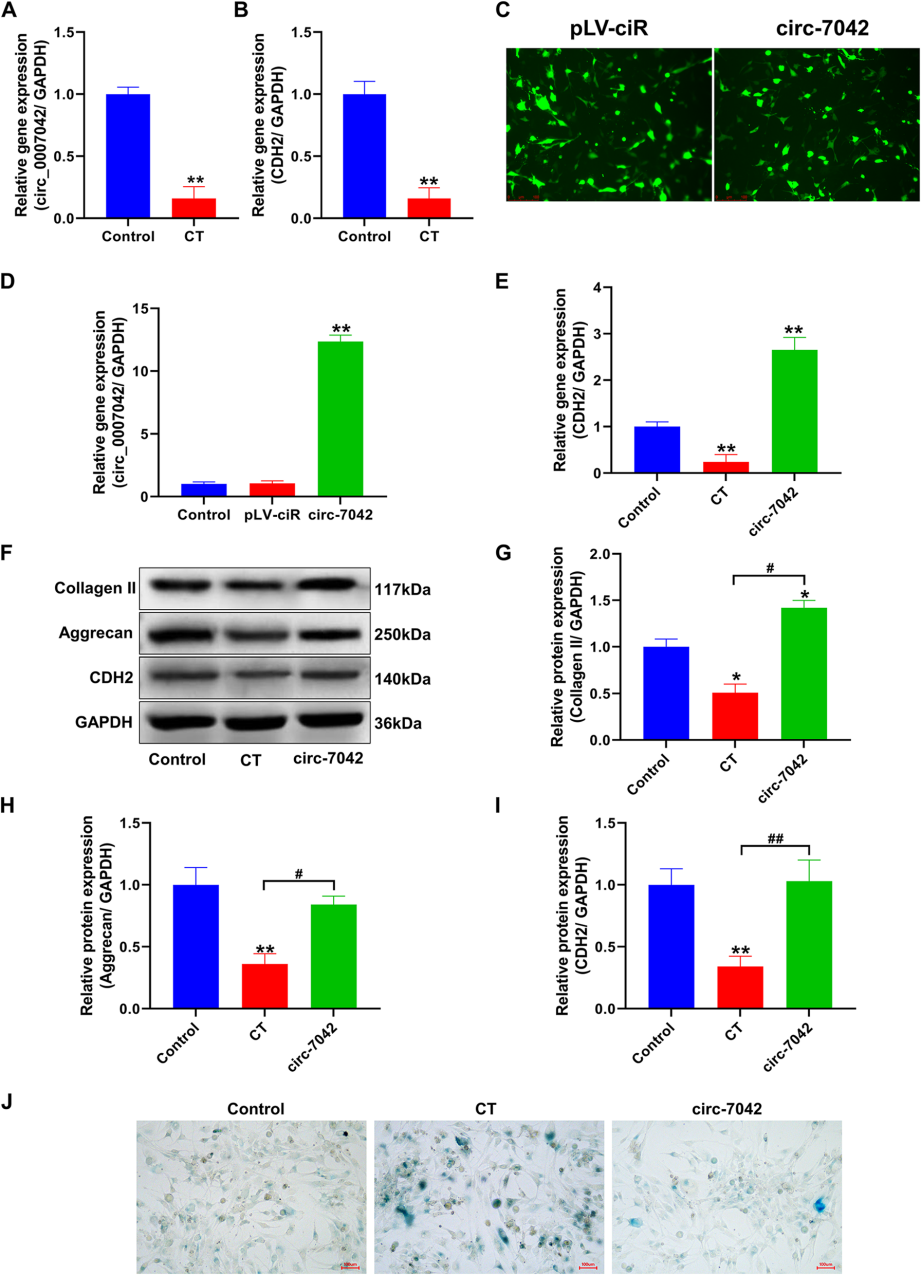
Effect of circ_7042 overexpression on NP cell function. **A** The expression level of the circ_7042 gene was detected by qPCR. **B** The expression level of the CDH2 gene was detected by qPCR. **C** Fluorescence microscopy of cells infected with lentivirus. **D** The expression level of the circ_7042 gene was detected by qPCR. **E** The expression level of the CDH2 gene in cells was detected by qPCR. **F** The expression levels of collagen II, aggrecan, and CDH2 proteins were detected by Western blotting. **G–I** Statistical quantification results of optical density values of protein bands detected by Western blotting. **J** The result of β-galactosidase staining. Blue cells represent senescent cells. *P<0.05, **P<0.01, compared with the control group. ^#^*P<*0.05, ^##^*P<*0.01, the two groups were compared between the wires

Figure 2A, B qPCR results show that the expression levels of the circ_7042 and CDH2 genes in the CT group were significantly decreased compared with those in the control group. The differences between the two groups were statistically significant (*P<*0.01). Subsequently, the pLV-circ-GFP-puro plasmid was used for circ_7042 overexpression. After plasmid construction and lentivirus packaging, NP cells were infected with lentivirus, as shown in Fig. 2C. Cells infected with vehicle virus and overexpressing circ_7042 emitted green fluorescence, indicating that the lentivirus successfully infected NP cells. qPCR was performed on cells infected with circ_7042 plasmid overexpression virus (Fig. 2D), and the results showed that the expression level of circ_7042 in the circ_7042 overexpression group was significantly increased, with statistically significant differences compared with the control and pLV-ciR groups (*P<*0.05). The qPCR results showed that the expression level of the CDH2 gene in NP cells overexpressing circ_7042 in the CT group was significantly higher than that in the vector control group (*P<*0.05, Fig. 2E). Combined with the results of Western blotting (Fig. 2F), overexpression of circ_7042 significantly reversed the decreases in collagen II, aggrecan, and CDH2 (*P<*0.05, Fig. 2G–I). To further investigate the effect of circ_7042 on cell senescence, β-galactosidase staining was performed to indicate senescent cells. As shown in Fig. 2J, senescent cells appeared blue. The results showed that the number of senescent cells in the CT group was significantly greater than that in the control group. In contrast, the number of senescent NP cells decreased significantly after overexpression of circ_7042.

### Overexpression of circ_7042 regulates NP cell apoptosis and collagen metabolism through the miR-369/CDH2 axis

**Fig. 3:**
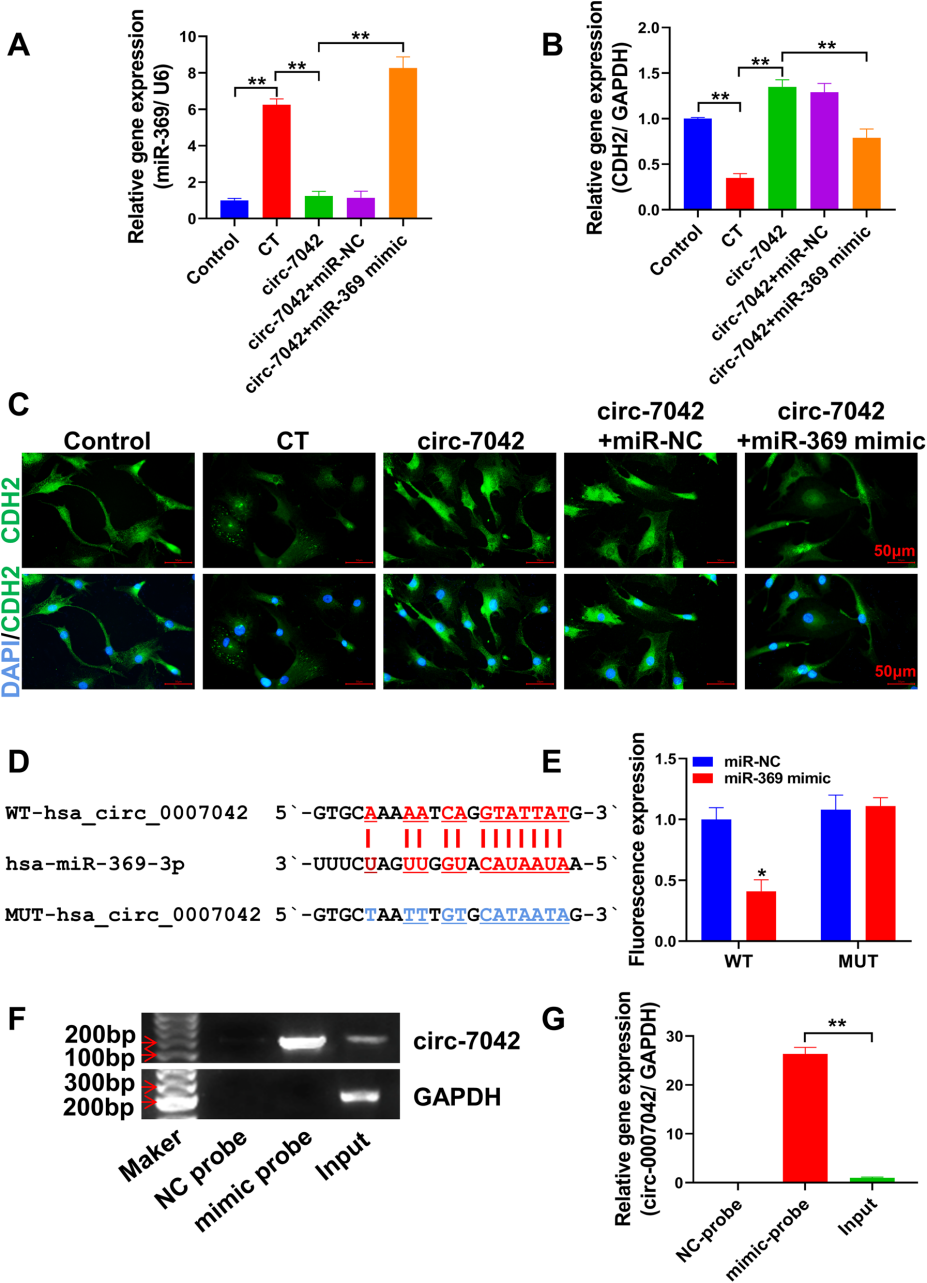
circ_7042 regulates miR-369-3p/CDH2. **A**, **B** The expression levels of the miR-369-3p and CDH2 genes were detected by qPCR. **C** The localization and expression level of CDH2 protein in NP were detected by immunofluorescence. **D** Targeted binding site of circ_7042 to miR-369-3p and mutation sequence of circ_7042. **E** Dual-luciferase activity; the fluorescence detection results were statistically quantified. **F** After miR-369 was adsorbed by RNA pull-down experiments, the circ_7042 gene was amplified by PCR, and agarose gel electrophoresis results were obtained. **G** The relative expression level of the circ_7042 gene in RNA enriched by the RNA pull-down assay was detected by qPCR. *P<0.05, ** *P<*0.01, the two groups were compared between the wires

Bioinformatics was used to predict the miRNA that circ_7042 might adsorb, combined with previous literature reports of miRNAs that could regulate CDH2 expression. After summarizing, it was found that the targeted binding sites of miR-369-3p overlapped with CDH2, and miR-369-3p overlapped with circ_7042. These findings provide a theoretical basis for the formation of the ceRNA regulatory network. Figure 3A shows that miR-369-3p gene expression levels were significantly elevated in the CT group (compared with the control group, *P<*0.05). Overexpression of circ_7042 significantly reversed the increase in miR-369-3p in NP cells under CT (compared with the CT group, *P<* 0.01). Figure 3B shows the expression level of CDH2 detected by qPCR. The results showed that the upregulation of the CDH2 gene in NP cells by circ_7042 was reversed after transfection with miR-369 mimic. Immunofluorescence (Fig. 3C) results also showed that the upregulation of CDH2 protein levels in NP cells by circ_7042 overexpression could be reversed after transfection with miR-369 mimic. Then, the interaction was determined by the luciferase reporter gene, as shown in Fig. 3D The predicted binding site between circ_7042 and miR-369-3p was present, and a mutation sequence was constructed for the targeted binding site. Figure 3E shows the dual-luciferase activity detection results. The fluorescence intensity in the WT group after cotransfection with circ_7042 WT and miR-369 mimic was significantly lower than that in the circ_7042 WT and miR-NC cotransfection group (*P<*0.05). No changes in fluorescence values were observed after transfection with miR-NC or miR-369 mimic in the MUT group. Combined with the RNA pull-down experiment, RNA enriched with miR-369 was amplified by PCR. Gel electrophoresis results (Fig. 3F) showed that a large amount of amplification could be observed in the mimic probe group of circ_7042. In contrast, no amplification of the circ_7042 gene was observed in the NC-probe group. The input group was unenriched lysate, and the circ_7042 gene amplification band could be observed. qPCR results also showed (Fig. 3G) that the expression level of the circ_7042 gene in the mimic probe group was significantly higher than that in the NC-probe and input groups (*P<*0.01).

**Fig. 4:**
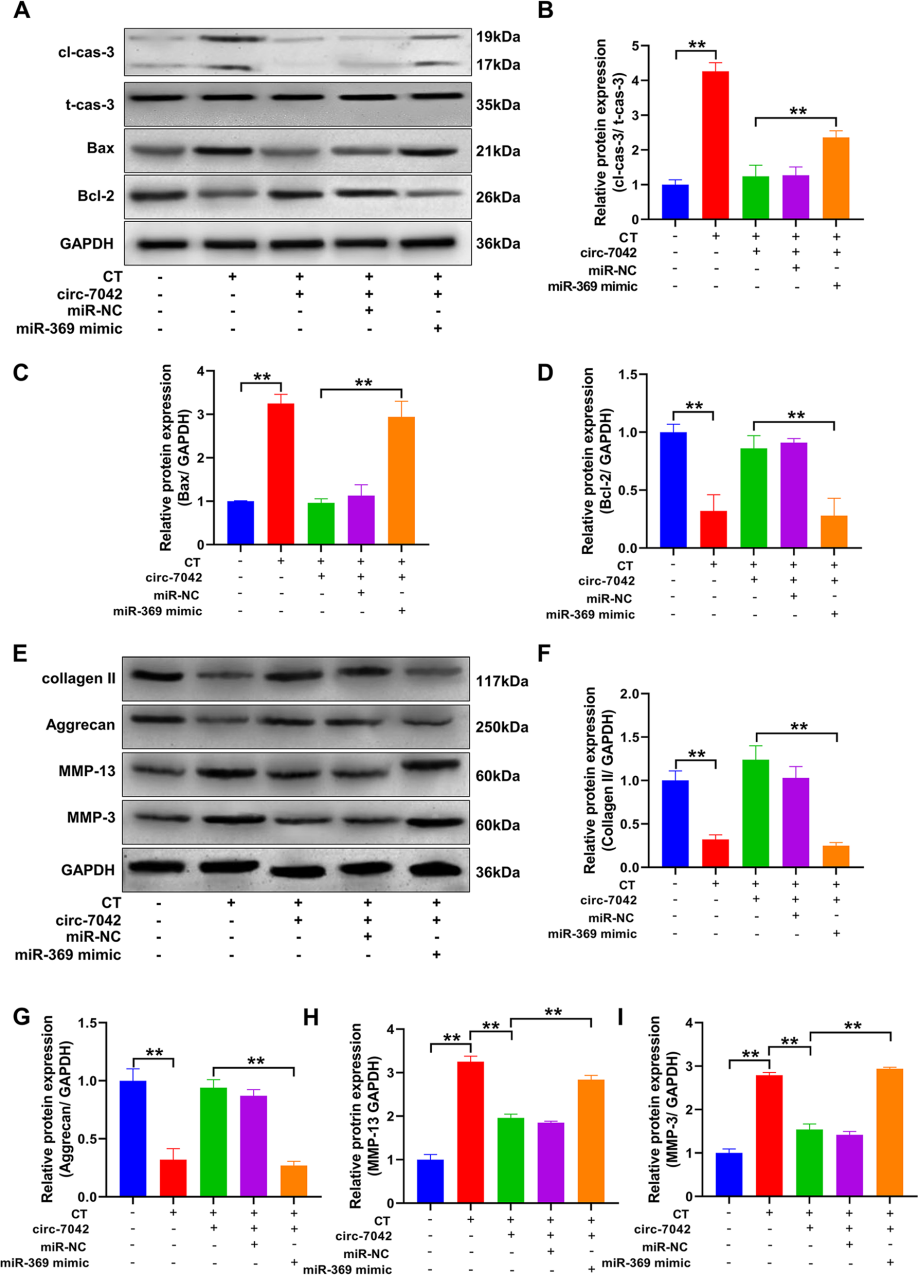
circ_7042 regulates NP cell apoptosis and collagen metabolism through miR-369-3p/CDH2. **A** Western blotting was used to detect the protein expression levels of cleaved caspase-3, total caspase-3, Bax and Bcl-2. **B–D** Statistical quantification results of cleaved caspase-3, total caspase-3, Bax, and Bcl-2 band optical density values in Western blot results in **A**. **E** Western blotting was used to detect the protein expression levels of collagen II, aggrecan, MMP-13, and MMP-3. **F–I** Statistical quantification results of optical density values of collagen II, aggrecan, MMP-13, and MMP-3 bands in the Western blotting results in **A**, respectively. *P<0.05, **P<0.01, the two groups were compared between the wires

Western blotting was used to detect apoptosis-related proteins (Fig. 4A). Statistical results showed (Fig. 4B–D) that, compared with the CT group, overexpression of circ_7042 significantly increased the protein expression level of Bcl-2 in NP cells after CT and significantly inhibited and reduced cleaved caspase-3 and Bax expression levels, with statistically significant differences between the two groups (*P<*0.05). The miR-369 mimic-cotransfected cells overexpressing circ_7042 could significantly reverse the increase in antiapoptotic proteins and the inhibition of proapoptotic proteins in NP cells. Western blotting was used to detect collagen metabolism-related proteins (Fig. 4E). The results showed that overexpression of circ_7042 increased the protein expression levels of collagen II and aggrecan in NP cells in a compression environment (Fig. 4F–G) and decreased the expression levels of the collagen degradation-related proteins MMP-13 and MMP-3. Compared with the CT group, the difference between the two groups was statistically significant (Fig. 4H, I, *P<*0.05). However, cotransfection of circ_7042-overexpressing cells with miR-369 mimic significantly reversed the increase in collagen formation protein in NP cells and the inhibition of collagen degradation-related proteins.

### Circ-7042 regulates NP cell apoptosis and collagen metabolism through miR-369-3p/BMP2

**Fig. 5:**
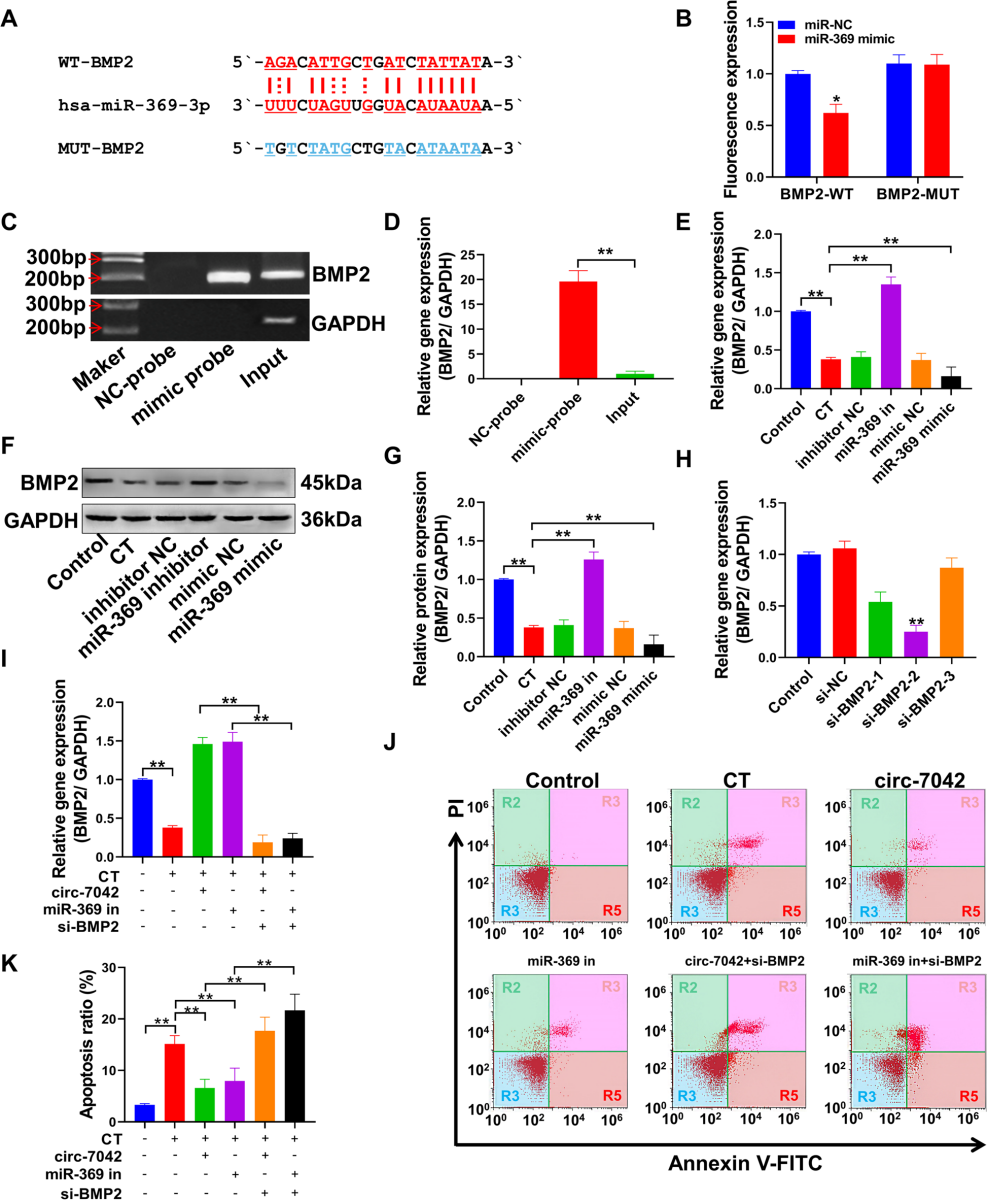
miR-369-3p regulates NP cell apoptosis through BMP2. **A** Targeted binding sequence and mutation sequence of miR-369-3p and BMP2-3′UTR. **B** Dual-luciferase activity and the statistical quantification results of fluorescence intensity. **C** After the RNA pull-down experiment, the RNA adsorbed by miR-369 was amplified by PCR, and agarose gel electrophoresis revealed the amplification band of the BMP2 gene. **D** The relative expression level of the BMP2 gene in the RNA pull-down experiment was detected by qPCR. **E** The relative expression level of the BMP2 gene after transfection with miR-369 mimic or inhibitor was detected by qPCR. **F** Western blotting was used to detect the relative expression level of BMP2 protein after transfection with miR-369 mimic or inhibitor. **G** Statistical quantification results of the optical density values of the immunoblotting bands. **H** The inhibitory effects of different siRNAs on the BMP2 gene were detected by qPCR. **I** qPCR verified the regulation of BMP2 gene expression levels by si-BMP2, circ-7042, and miR-369 inhibitor. **J** Apoptosis levels were measured by flow cytometry with the Annexin V-FITC fluorescence intensity on the horizontal axis and the PI staining fluorescence intensity on the vertical axis. R2 represents necrotic cells, R3 represents late apoptotic cells, R4 represents normal cells, and R5 represents early apoptotic cells. **K** The apoptosis level was detected by flow cytometry, and the percentage of apoptotic cells was statistically quantified. *P<0.05, **P<0.01, the two groups were compared between the wires

Figure 5A shows the targeted binding sites of the BMP2-3′UTR sequence with miR-369-3p and the sequences of the mutation sites of the BMP2-3′UTR. Figure 5B shows the dual-luciferase activity results, suggesting that the fluorescence detection intensity of the BMP2-WT group after cotransfection with miR-369 mimic and BMP2-3′UTR plasmid was significantly lower than that of the group cotransfected with miR-NC and BMP2-3′UTR plasmid. The difference between the two groups was statistically significant (*P<*0.05). Figure 5C shows the PCR amplification of miR-369-enriched RNA after RNA pull-down experiments and gel electrophoresis results. The BMP2 gene was amplified in the mimic probe group but not in the NC-probe group. BMP2 gene amplification bands were observed in the input group. qPCR results also showed (Fig. 5D) that the expression level of the BMP2 gene in the mimic probe group was significantly higher than those in the NC-probe and input groups (*P<*0.01). The expression level of the BMP2 gene in NP cells was significantly inhibited after CT, and transfection with miR-369 inhibitor significantly reversed the inhibitory effect of CT on BMP2 in NP cells. Transfection with the miR-369 mimic intensified the inhibition of BMP2 gene expression after CT (Fig. 5E). Verification was performed at the protein level (Fig. 5F, G), and the results were the same as those obtained at the gene level.

Therefore, the siRNA sequence was designed according to the CDS of BMP2. Among the three designed siRNAs, siRNA2 had the most significant inhibitory effect on the expression of the BMP2 gene, and the difference was extremely significant compared with the control group (*P<*0.01). Therefore, siRNA2 was used as BMP2 in subsequent experiments, denoted as si-BMP2. Then, we studied the relationship between circ_7042, miR-369 inhibitor, and si-BMP2 (Fig. 5H). The qPCR results showed (Fig. 5I) that transfection of si-BMP2 reversed the upregulation of circ_7042 and miR-369 inhibitor on the BMP2 gene. Figure 5J shows the detection of cell apoptosis levels by flow cytometry. The results revealed that both circ_7042 and miR-369 inhibitor reduced the apoptosis level of NP cells under CT, and the difference was statistically significant compared with the CT group (*P<*0.05). Si-BMP2 transfection significantly reversed the effect of circ_7042 and miR-369 inhibitor on NP cell apoptosis (Fig. 5K, *P<*0.05).

**Fig. 6:**
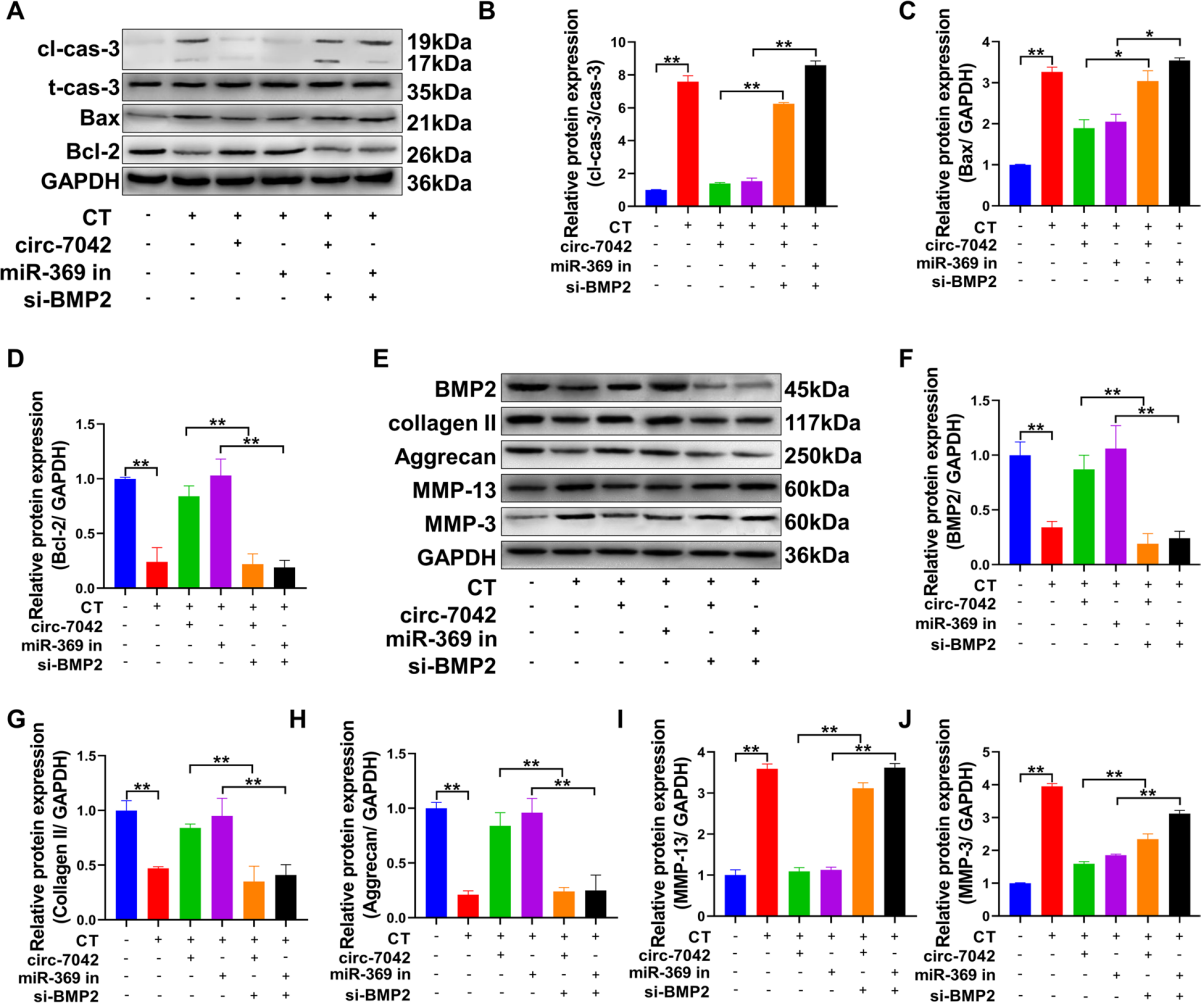
circ_7042 regulates the expression of apoptosis- and collagen metabolism-related proteins in NP cells through the miR-369-3p/BMP2 axis. **A** Western blotting was used to detect cleaved caspase-3, total caspase-3, Bax, and Bcl-2 protein expression levels. **B–D** The optical density of the protein bands in A was statistically quantified by Western blot detection. **E** The expression levels of BMP2, collagen II, aggrecan, MMP-13, and MMP-3 proteins were detected by Western blotting. **F–J** Western blotting results: statistical quantification results of optical density values of protein bands in **E**. *P<0.05, **P<0.01, the two groups were compared between the wires

Western blotting was used to detect the apoptosis level of cells (Fig. 6A–D). Si-BMP2 transfection significantly reversed the inhibitory effect of circ_7042 and the miR-369 inhibitor on the proapoptotic proteins cleaved caspase-3 and Bax in NP cells and the promoting effect of the antiapoptotic protein Bcl-2, with a statistically significant difference (*P<*0.05). Meanwhile, BMP2 and collagen metabolism-related proteins were detected by Western blotting (Fig. 6E–J). The results indicate that si-BMP2 transfection can significantly reverse the inhibitory effect of circ_7042 and miR-369 inhibitor on the collagen-degrading proteins MMP-13 and MMP-3. In addition, it also changed the promoting effects of circ_7042 and miR-369 inhibitor on collagen II, aggrecan, and BMP2 proteins, and the differences between groups were statistically significant (*P<*0.05).

### circ_7042 slows the senescence of NP cells through the BMP2/PI3K/Akt pathway

**Fig. 7:**
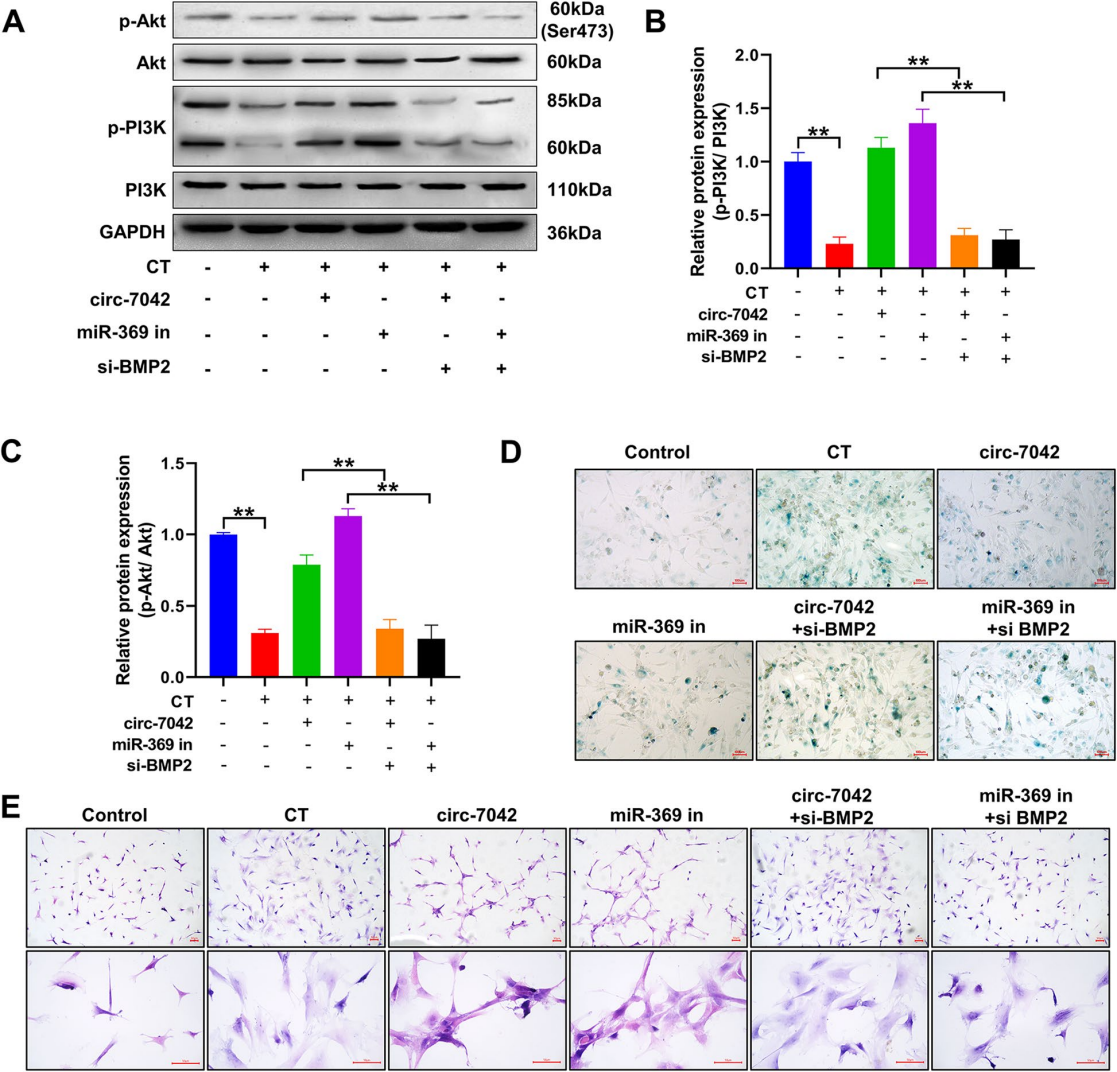
circ_7042 slows the senescence of NP cells through the BMP2/PI3K/Akt pathway. **A** Western blotting was used to detect the phosphorylation level of the PI3K/Akt signalling pathway. **B**, **C** Statistical quantification results of optical density values of Western blotting protein bands. **D** β-galactosidase staining, with senescent cells shown in blue. **E** HE staining results. *P<0.05, **P<0.01, the two groups were compared between the wires

Figure 7A shows the detection of PI3K/Akt pathway proteins and phosphorylation by Western blotting. The results showed (Fig. 7B, C) that the phosphorylation of PI3K/Akt in NP cells was significantly inhibited after CT (compared with the control group, *P<*0.05). Transfection with circ-7042 or miR-369 inhibitor effectively increased the PI3K/Akt phosphorylation level of NP cells. Nevertheless, this effect was reversed after transfection with si-BMP2, and the difference between groups was statistically significant (*P<*0.05). Figure 7D shows the results of β-galactosidase staining. Inhibition of BMP2 expression can reverse the slowing effect of circ_7042 and miR-369 inhibitor on NP cell senescence. Figure 7E shows the results of HE staining. After transfection with circ_7042 or miR-369 inhibitor, the change in NP cell morphology was alleviated, with abundant cytoplasm and no significant cell enlargement. si-BMP2 reversed the beneficial effects of circ_7042 or miR-369 inhibitor on cell morphology.

### Protective effect of circ_7042 on intervertebral discs in LSI mice

**Fig. 8:**
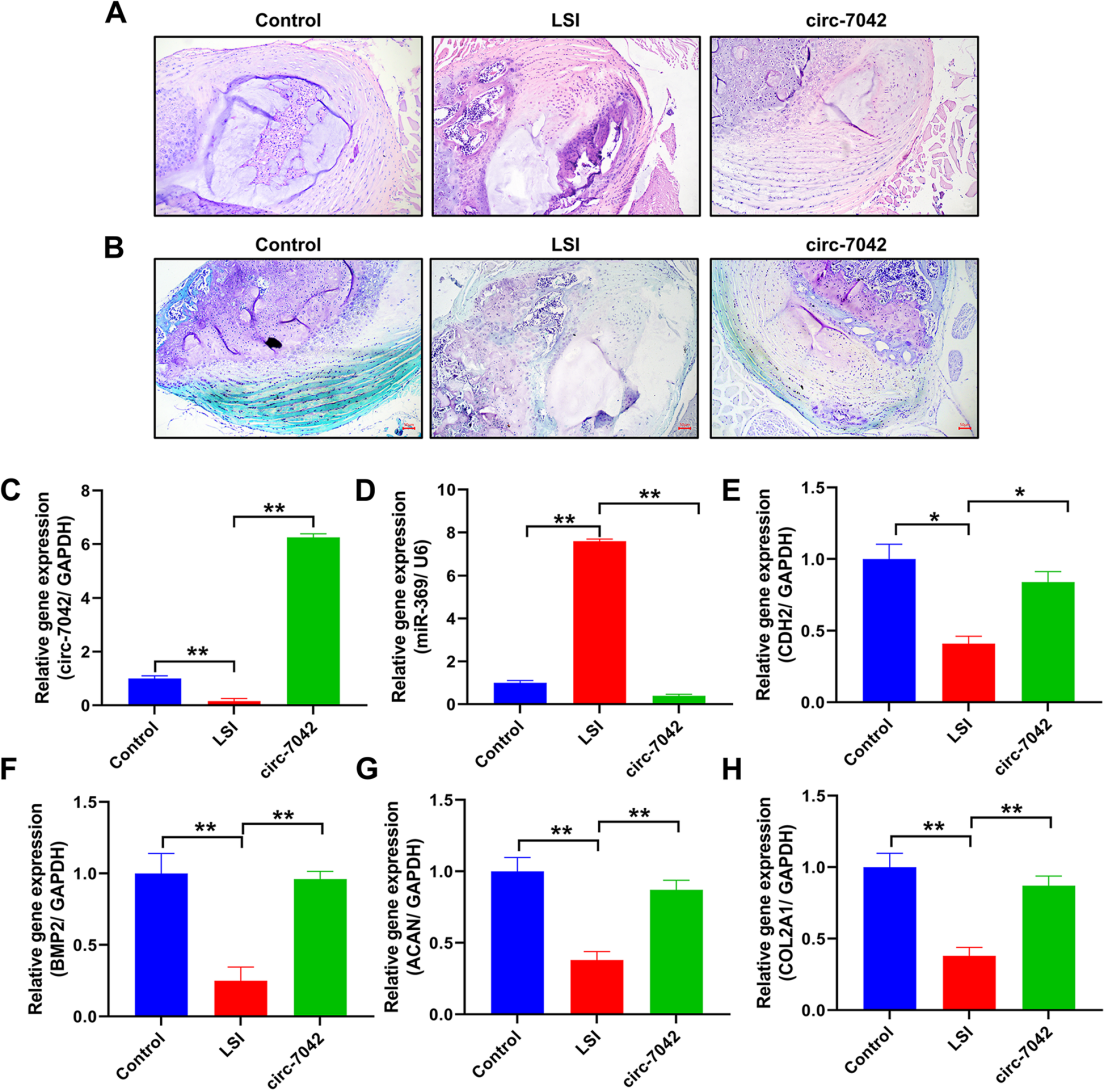
Protective effect of circ_7042 on intervertebral discs in LSI mice. **A** HE staining results of intervertebral disc nucleus pulposus tissue in mice. **B** Safranin O-bright green staining results; red is cartilage and glycoprotein, and green is collagen fibre. **C–H** The expression levels of circ_7042, miR-369-3p, Cdh2, Bmp2, Acan, and Col2a1 were detected by qPCR. *P<0.05, **P<0.01, the two groups were compared between the wires

Figure 8A shows the results of HE staining for intervertebral disc tissue. There were many NP cells in the NP of the control group, and the annulus fibrosus tissue was typically arranged. Conversely, the NP cells in the LSI group were lost, and the annulus fibrosus showed apparent rupture and tissue destruction. After the administration of circ_7042, the loss of NP cells was also observed, but the structure of the annulus fibrosus was standard, without apparent damage. Figure 8B shows safranine O-bright green staining with red–purple proteoglycan positivity and green collagen fibre positivity. In the LSI group, the staining of the cartilage tissue and NP was weak. In contrast, the positive level of the staining was higher, and glycoprotein loss was reduced after circ_7042 adenovirus injection. In LSI mice (Fig. 8C–H), the gene expression levels of circ_7042, miR-369, Cdh2, Bmp2, Acan, and Col2a1 were significantly decreased, while the gene expression levels of miR-369-3p were increased considerably, with statistically significant differences compared with the control group (*P<*0.05). After circ_7042 adenovirus injection, the gene expression levels of circ_7042, Cdh2, Bmp2, Acan, and Col2a1 were significantly increased, while the gene expression levels of miR-369-3p were significantly inhibited, with statistically significant differences compared with the LSI group (*P<*0.05).

**Fig. 9:**
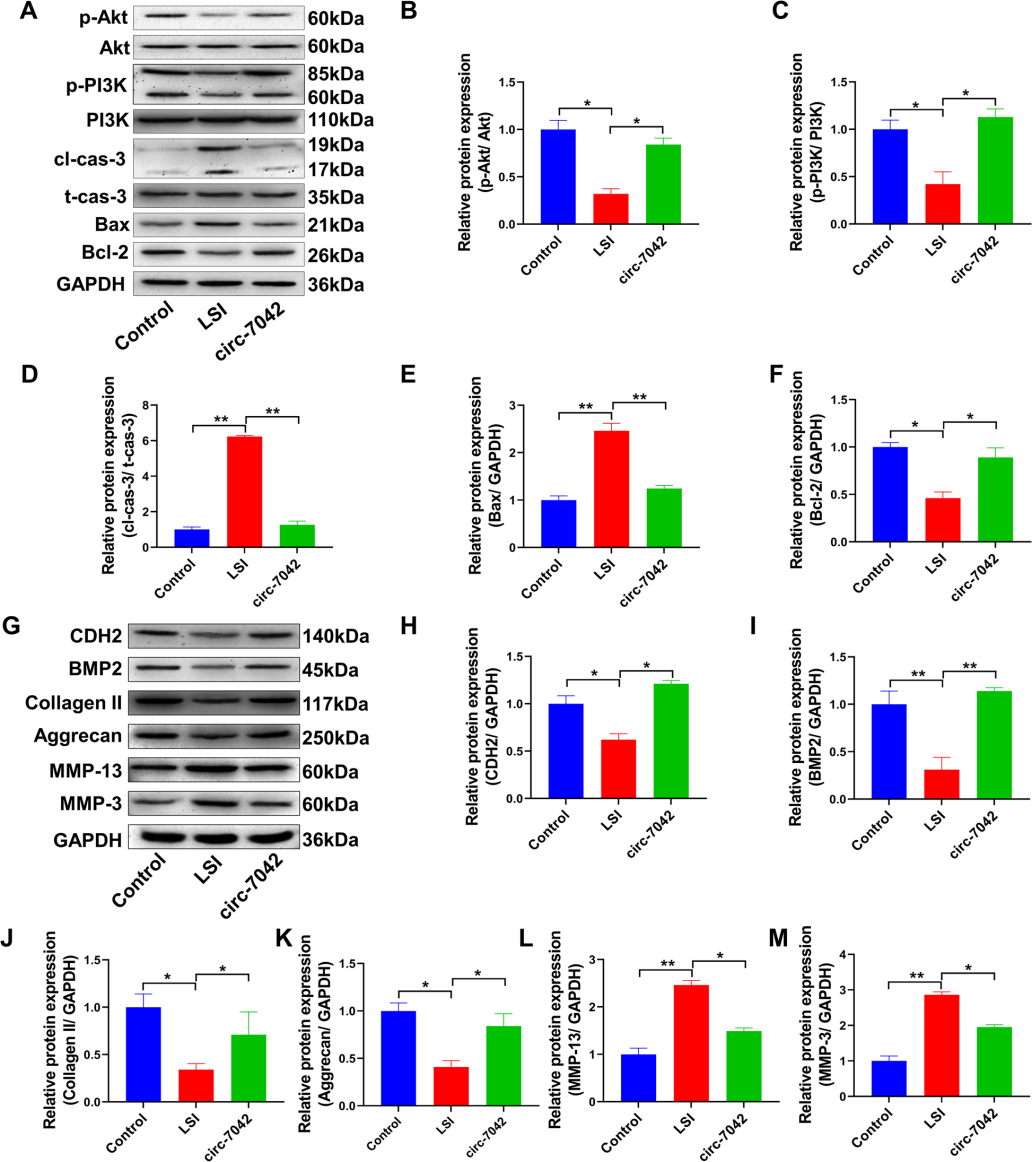
circ_7042 inhibits LSI-induced NP cell apoptosis and collagen degradation through the BMP2/PI3K/Akt pathway. **A** Western blotting was used to detect PI3K/Akt protein and phosphorylation levels and Bcl-2, Bax, total caspase-3, and cleaved caspase-3 protein expression levels. **B–F** The optical density of the immunoblotting protein bands was statistically quantified. **G** The expression levels of CDH2, BMP2, collagen II, aggrecan, MMP-13, and MMP-3 proteins were detected by Western blotting. **H–M** Statistical quantification results of optical density values of Western blotting protein bands. *P<0.05, **P<0.01, the two groups were compared between the wires

Western blotting was used to detect apoptosis and phosphorylation of the PI3K/Akt pathway (Fig. 9A). The results showed that cleaved caspase-3 and Bax protein expression levels were significantly increased, while antiapoptotic Bcl-2 protein expression levels were decreased in LSI model mouse NP tissues (Fig. 9B–F). No significant changes were observed in total protein levels of PI3K/Akt, but the phosphorylation levels were significantly abolished (compared with the control group, *P<*0.05). circ_7042 significantly reduced the expression levels of the proapoptotic proteins cleaved caspase-3 and Bax in LSI mice but upregulated the expression of the antiapoptotic protein Bcl-2 and upregulated the phosphorylation level of the PI3K/Akt pathway (compared with the LSI group, *P<*0.05). As shown in Fig. 9G–M, compared with the control group, the expression levels of CDH2, BMP2, collagen, and aggrecan proteins in the LSI group were significantly inhibited, while the expression levels of MMP-13 and MMP-3 proteins were increased considerably. The difference between groups was statistically significant (*P<*0.05). Compared with the LSI group, the protein expression levels of CDH2, BMP2, collagen, and aggrecan in the circ_7042 group were significantly increased, and the protein expression levels of MMP-13 and MMP-3 were significantly inhibited, with statistically significant differences between groups (*P<*0.05).

## Discussion

To date, IDD has limited therapeutic alternatives and far from satisfactory clinical efficacy, which is highly related to the unclear pathogenesis of IDD. Therefore, further exploration of the pathogenesis of IDD is crucial for the development of accurate pathophysiological diagnosis and the formulation of precise treatment plans [19, 20]. Intervertebral discs (IVDs) are subjected to a large amount of mechanical load in the body, which plays a vital role in the development of IDD [21]. The abnormal mechanical load has been shown to induce various pathological changes in IVDs, including the two leading causes of IDD, namely, NP cell apoptosis and ECM destruction, which impair the physiological function of IVDs [22, 23].

It is well known that mechanical stress is a significant cause of IDD, and therefore, the conditions that can cause the loss of strength and frequency of mechanical stress of IDD are crucial. Studies have shown that static and continuous stress conditions can cause NP cell degeneration [24]. Moreover, mitochondria-dependent apoptosis of NP cells was observed under such simulated experimental conditions [25]. Therefore, mechanical static compression was used to simulate the process of IDD caused by compression injury of the human intervertebral disc.

Due to the previous proof that CDH2 can slow the senescence of NP cells under compression induction [6], this study mainly focuses on the ncRNA network that regulates CDH2 regulation. Through bioinformatics prediction and differential gene expression in human tissue samples, we determined that hsa_circ_7042 and miR-369-3p may jointly play a regulatory role in the CDH2 gene.

The construction of hsa_circ_7042-overexpressing NP cells was further verified. The results showed that after CT, the expression of ECM catabolic enzymes (MMP-3 and MMP-13) and proapoptotic genes (Bax and cleaved caspase-3) in NP cells increased, and the expression of ECM anabolic markers (collagen II and aggregation protein) and the antiapoptotic protein Bcl-2 decreased. Moreover, the expression level of CDH2 protein was also significantly increased, and the changes in these indices were significantly reversed by overexpression of hsa_circ_7042 in NP cells. IVDs are the largest vascular minor organs in the body, consisting of a central colloidal NP and a surrounding annulus fibrosus (AF) [26]. NP cells are the primary resident cells in highly hydrated NP tissues and are responsible for controlling the synthesis and decomposition of the NP extracellular matrix (ECM) and maintaining the standard structure and functional characteristics of IVDs [27]. When the ECM catabolism of NP cells exceeded internal anabolism, aggrecan and collagen II were degraded, resulting in the dehydration and absorption of NP; additionally, the height of the intervertebral disc decreased, and the ability to resist IDD decreased [28]. IDD is marked by the progressive loss of ECM macromolecules, proteoglycans, and type II collagen. The loss of type II collagen is an early sign of IDD [29]. Aggrecan is a significant proteoglycan in NP tissue that is essential for normal disc function [30].

Based on the above characteristics and the differential expression of aggrecan and collagen II proteins, it can be determined that overexpression of hsa_circ_7042 can reduce the apoptosis of NP cells caused by continuous static CT degradation of ECM and improve the expression of CDH2. The dual-luciferase activity of miR-369-3p was reported, and an RNA pull-down assay was performed. The results proved that miR-369-3p and hsa_circ_7042 are indeed in a targeted binding relationship. However, current knowledge about the biological role of hsa_circ_7042 in IDD integrity remains limited and vague. To our knowledge, no studies have shown that hsa_circ_7042 and miR-369-3p interact with miRNAs in human NP cells to regulate apoptosis and ECM degradation.

When the preliminary information related to disc degeneration in the Genecard database was combined with the target analysis of miR-369-3p, it was found that CDH2 was not included in the database, and the BMP2 gene was the primary target gene obtained by screening. As microRNAs (miRNAs) are small and highly conserved endogenous ncRNAs, most of them regulate posttranscriptional gene expression in mammals by targeting the mRNA 3′-untranslated region (3′-UTR) [13]. It has also been suggested that a single miRNA may target multiple mRNAs, which jointly regulate the exact molecular or cellular processes [31, 32]. We hypothesized that the terminal gene of miR-369-3p that plays a functional role in disc degeneration might also pass through BMP2 in addition to CDH2. Therefore, we combined dual-luciferase activity and RNA pull-down experimental data for verification and confirmed the targeted binding relationship between miR-369-3p and BMP2. BMP2 is a group of BMPs belonging to the TGF-β family that play crucial roles in bone development and repair [33].

BMP2 can directly promote collagen synthesis by stimulating chondrocytes [34]. Therefore, BMP2 is likely to be another critical end effector mRNA of hsa_circ_7042 and miR-369-3p in regulating disc degeneration. In the NP cell degeneration model, overexpression of hsa_circ_7042 and inhibition of miR-369-3p both led to increased expression of the BMP2 gene and protein.

Based on these findings, we designed an siRNA targeting BMP2. We demonstrated that inhibition of BMP2 can effectively reverse the protective effects of hsa_circ_7042 and miR-369 inhibitors on intervertebral disc NP and enhance the expression levels of proapoptotic and ECM degradation-related proteins. These results were consistent with the experiment of Tan [35] and Daniel [36], which showed that shBMP2 treatment could significantly upregulate collagen II and aggrecan in NP cells and inhibit the protein expression of MMP-13. Analysis of downstream regulatory proteins of BMP2 showed that BMP2 plays a vital role in activating the PI3K/Akt pathway in antiapoptosis and promoting the proliferation of NP cells. For example, Tan et al. [35] suggested that BMP2 inhibits apoptosis by upregulating the phosphorylation level of the PI3K/Akt signalling pathway. BMP2 induces actin cytoskeleton reorganization and cell migration by activating the PI3K/Akt pathway [37]. In addition, other studies have indicated that CDH2 can attenuate NP cell apoptosis induced by high-intensity compression by activating PI3K/Akt-GSK-3β signalling [38]. The activated PI3K/Akt pathway helps prevent ECM degradation in IDD, inhibit NP cell apoptosis, and promote cell proliferation [39]. Therefore, we believe that the expression levels of CDH2 and BMP2 are upregulated after the absorption of miR-369-3p by the hsa_circ_7042 sponge, thereby activating the PI3K/Akt pathway. In subsequent validation, we confirmed that overexpression of hsa_circ_7042 upregulated the expression levels of CDH2 and BMP2 proteins and increased the phosphorylation level of PI3K/Akt.

For further verification in animals, we constructed LSI mice to construct a model of mechanical cell compression [21]. Downregulation of circ_7042 and significant upregulation of miR-369-3p gene levels were detected in LSI mice. ECM protein synthesis was decreased, degradation was increased, and apoptosis levels were increased in the NP tissues of the LSI mice. These findings are consistent with the model research results of Zheng [17] et al. After intervertebral disc injection of circ_7042 packed with adenovirus, CDH2 and BMP2 gene and protein expression levels in NP tissues increased, while the apoptosis level decreased. The symptoms of pathological injury were relieved, the structure of the annulus fibrosus was obviously improved, and the loss of glycan protein in cartilage tissue was significantly reduced.

## Conclusion

In summary, previous studies have shown that CDH2 plays a protective role in IDD, and bioinformatics technology was used to predict and screen the regulatory network of CDH2 ncRNA. The hsa_circ_7042/miR-369-3p axis was determined through targeted prediction and high-throughput sequencing results, and then the reverse clues were used to predict and screen the factors related to miR-369-3p and disc degeneration. The new mechanism of hsa-circ_7042/miR-369-3p/BMP2 was identified, and the PI3K/Akt pathway of CDH2 and BMP2 interaction was verified. In cell and animal experiments, it was confirmed that overexpression of hsa_circ_7042 could inhibit the gene expression level of miR-369-3p, upregulate the expression levels of CDH2 and BMP2, activate the PI3K/Akt pathway, and enhance the resistance effect of NP cells to IDD.

## Supplementary Information

**Additional file 1: Table S1.** List of the PCR Primer Pairs chosen for *in vitro* PCR validation. **Table S2.** List of the Hsa_circ_7042 overexpressed primer. **Table S3.** List of BMP2 siRNA primers. **Table S4.** Plasmid construction primers involved in the dual-luciferase activity reporting experiment.

## Data Availability

The datasets used and/or analysed during the current study are available from the corresponding author on reasonable request.

## Abbreviations

IDD: Intervertebral disc degeneration
ECM: Extracellular matrix
NP: Nucleus pulposus
CDH2: N-cadherin
CT: Compression treatment
IVD: Intervertebral disc
BMP2: Bone morphogenetic protein 2
