# Supplementary material for: Circ0007042 alleviates intervertebral disc degeneration by adsorbing miR-369 to upregulate BMP2 and activate the PI3K/AKt pathway
Source: Arthritis Res Ther. 2022 Sep 6;24:214. doi: 10.1186/s13075-022-02895-7 (PMC9446735; doi:10.1186/s13075-022-02895-7)
Supplement: Supplementary file 1 — Additional file 1: Table S1. List of the PCR Primer Pairs chosen for in vitro PCR validation. Table S2. List of the Hsa_circ_7042 overexpressed primer. Table S3. List of BMP2 siRNA primers. Table S4. Plasmid construction primers involved in the dual-luciferase activity reporting experiment. [file 13075_2022_2895_MOESM1_ESM.doc]

### Table 1. List of the PCR Primer Pairs chosen for in vitro PCR validation

| Name | Forward primer | Reverse primer |
| --- | --- | --- |
| hsa_circ_7042 | 5′-GGGATGATAACGCCAAAACAGG-3′ | 5′-CCTTCGAGGCTGCTTCTAACA-3′ |
| CDH2 | 5′-CCTCCAGAGTTTACTGCCATGAC-3′ | 5′-GTAGGATCTCCGCCACTGATTC-3′ |
| BMP2 | 5′-TGTATCGCAGGCACTCAGGTCA-3′ | 5′-CCACTCGTTTCTGGTAGTTCTTC-3′ |
| GAPDH | 5′-GTCTCCTCTGACTTCAACAGCG-3′ | 5′-ACCACCCTGTTGCTGTAGCCAA-3′ |
| hsa-miR-369-5p | 5′-TCGACCGTGTTATATTCG-3′ | 5′-GAACATGTCTGCGTATCTC-3′ |
| U6 | 5′-CTCGCTTCGGCAGCACAT-3′ | 5′-TTTGCGTGTCATCCTTGCG-3′ |
| mmu_circ_7042 | 5′-ACCGACTTCGATGGGGTAGA-3′ | 5′-GAAACGATGACGGCGTTCAG-3′ |
| Acan | 5′-CAGGCTATGAGCAGTGTGATGC-3′ | 5′-GCTGCTGTCTTTGTCACCCACA-3′ |
| Col2a1 | 5′-GCTGGTGAAGAAGGCAAACGA G-3′ | 5′-CCATCTTGACCTGGGAATCCAC-3′ |
| Gapdh | 5′-CATCACTGCCACCCAGAAGACTG-3′ | 5′-ATGCCAGTGAGCTTCCCGTTCAG-3′ |
| mmu-miR-369-5p | 5′-GATCGACCGTGTTATATTCG-3′ | 5′-GAACATGTCTGCGTATCTC-3′ |
| mmu-U6 | 5′-AGGACGACAGTGCAAAGCATGG-3′ | 5′-CAGCAATGCGTCAAGCAGATC C-3′ |

### Table 2. List of the Hsa_circ_7042 overexpressed primer

|  | Primer |
| --- | --- |
| Exon 2-F-MluI | 5′-GTTCACGCGTTTTCCTCCCTCGCCCTGCAGAGTAACCTGATCCACTACC-3′ |
| Exon 3-R-XhoI | 5′-CGGTCTCGAGGACTAGCATTAATTACTTACTCAATGGAAACAATGACAGC-3′ |

### Table 3. List of BMP2 siRNA primers

|  | SS-Primer | AS-Primer |
| --- | --- | --- |
| siRNA1 | 5′-UGUUUCAGGCCGAACAUGCUG-3′ | 5′-GCAUGUUCGGCCUGAAACAGA-3′ |
| siRNA2 | 5′-UCGUUUCUGGUAGUUCUUCCA-3′ | 5′-GAAGAACUACCAGAAACGAGU-3′ |
| siRNA3 | 5′- UCAUCUUGGUGCAAAGACCUG-3′ | 5′-GGUCUUUGCACCAAGAUGAAC-3′ |

### Table 4. Plasmid construction primers involved in the dual-luciferase activity reporting experiment

|  | Forward primer | Reverse primer |
| --- | --- | --- |
| Circ_7042-WT | ScaI-5′-AAAAGTACTGTGCAAAAATC  AGGTATTATG-3′ | XbaI-5′-TGCTCTAGACTGATAGATTAAA  TGGTATA-3′ |
| Circ_7042-MUT | ScaI-5′-AAAAGTACTGTGCTAATTTGT  GCATAATAG-3′ | XbaI-5′-TGCTCTAGACTGATAGATTAAA  TGGTATA-3′ |
| BMP2 WT | ScaI-5′-AAAAGTACTTGTCTATGCTGT ACATAATAA-3′ | XbaI-5′-TGCTCTAGATACTTTGGCCAGA  TCAGCCC-3′ |
| BMP2-MUT | ScaI-5′-AAAAGTACTTGTCTATGCTGT ACATAATAA-3′ | XbaI-5′-TGCTCTAGATACTTTGGCCAGA  TCAGCCC-3′ |
